# Genotoxicity Comparison between *Morinda citrifolia* Fruit and Seed Substances

**DOI:** 10.3390/foods11121773

**Published:** 2022-06-16

**Authors:** Sarah Shin, Ji Soo Kim, Myung Ku Park, Ok-Sun Bang

**Affiliations:** 1 KM Science Research Division, Korea Institute of Oriental Medicine, 1672 Yuseong-daero, Yuseong-gu, Daejeon 34054, Korea; s.sarah@kiom.re.kr; 2 Korea Testing & Research Institute, Hwasun 58141, Korea; js4103@ktr.or.kr (J.S.K.); bluesky@ktr.or.kr (M.K.P.)

**Keywords:** aqueous extract, fruit, genotoxicity, *Morinda citrifolia* L. *(Rubiaceae)*, noni, powder, puree

## Abstract

This study aimed to evaluate the genotoxic potential of the fruit and seed powder, fruit puree, and aqueous extracts of *Morinda citrifolia* (*Rubiaceae,* noni). The genotoxic potential of the noni substances was evaluated using in vitro Ames, in vitro chromosomal aberration, and in vivo micronucleus tests. All test procedures were conducted per Organization for Economic Cooperation and Development guidelines, and good laboratory practice. None of the noni fruit test substances showed genotoxic signs up to 5000 and 2000 μg/plate in the Ames and micronucleus tests, respectively. In the chromosomal aberration test, neither the fruit puree nor aqueous extract showed structural and numerical aberrations up to 5000 and 4650 μg/mL, respectively, irrespective of metabolic activation, in both 6 h and 24 h treatment groups. The safe ranges of noni fruit and seed powders were up to 2500 and 2100 μg/mL, respectively, in the 6 h treatment group and up to 1600–4100 and 370–450 μg/mL, respectively, in both 6 h and 24 h treatment groups in the presence of metabolic activation. Noni fruit and seeds were safe in terms of genotoxicity under our experimental conditions. Our data are the first to provide valuable genotoxic information on *Morinda citrifolia*.

## 1. Introduction

*Morinda citrifolia* Linn. is a member of the genus *Morinda* in the family *Rubiaceae*. It is commonly called noni, the Hawaiian name, and is also called Indian mulberry, Mengkudu, or cheese fruit [1,2]. Noni is distributed in southern Asia, the Pacific region, the Caribbean, and Africa. Noni is a perennial evergreen tree, and its fruit is approximately 5–10 cm in length and 3–6 cm in width and contains up to 260 seeds [3,4]. Noni fruits have traditionally been used to treat mouth sores, cancer, and urinary and gastrointestinal tract disorders such as stomach ulcers, diarrhea, tuberculosis, and bacterial and fungal infections [5,6,7]. Current studies have reported that noni fruits exhibit antioxidant, antiarthritic, and immunity-enhancing activities and prevent metabolic diseases [8,9,10,11]. Recent phytochemical studies of noni fruit have demonstrated that damnacanthal, xeronine, and polyssaccharide exert anticancer activity; deacetylasperulosic acid (DAA) has in vivo antioxidant and anti-genotoxic activities; and scopoletin exerts analgesic properties, anti-obesity activity, and anti-inflammatory activity (reviewed in [2,4,12,13].

Since the European Union approved the commercial source of noni fruit juice from French Polynesia as a novel food, noni fruits are widely used as dietary supplements in various forms such as puree, juice, pills, aqueous extracts, and powder [5,6,11,12,13,14]. Many publications describe the various health benefits of noni features including fruit, leaf, stem, and seeds; however, reports on their safety are limited [6,12,13]. It has been reported that regular intake of noni juice does not cause any toxic effects, including acute toxicity, hepatotoxicity, or sub-chronic toxicity [15,16,17,18,19,20]. Despite the worldwide use and pharmacological significance of noni fruit, its genotoxicity has not been clearly elucidated. Several studies have demonstrated the genotoxicity of other traditionally used plants, such as *Cynara scolymus* L. [21,22,23]. In addition, several studies have shown that anthraquinones may exert genotoxicity and carcinogenicity [24,25], and anthraquinones such as lucidin, alizarin, and rubiadin have been found in noni fruits, leaves, and roots [15,26]. Therefore, the genotoxic potential of noni preparations cannot be excluded considering their content, and the genotoxicity assessment or mutational effects could minimize the possible risks of noni fruits to human health.

Genotoxicity tests of noni juice did not show any genotoxic potential in a battery of short-term tests using the *Salmonella* microsome assay and the mammalian mutagenicity test [20,27]. Rather, noni juice reduced mitomycin C and doxorubicin-induced genotoxicity in somatic cells of Drosophila melanogaster [28]. However, current genotoxicity assessment under Organization for Economic Cooperation and Development (OECD) guidelines in effect today include the in vitro Ames test, in vitro mammalian chromosomal aberration test, and in vivo mammalian erythrocyte micronucleus test. Therefore, in order to provide information on the genotoxic potential of noni fruit and seeds, in this study, we conducted genotoxicity tests on noni products, including a puree of noni fruit (PNF), an aqueous extract of dried noni fruit (EDNF), a powder of dried noni fruit (PDNF), and a powder of dried noni seed (PDNS).

## 2. Materials and Methods

### 2.1. Chemicals and Reagents

Authentic reference chemicals (deacetylasperulosidic acid, DAA; asperulosidic acid, AA; and 5-Hydroxymethylfurfural, 5-HMF) were obtained from ChemFaces (Wuhan, China). High performance liquid chromatography (HPLC)-grade solvents and analytical-grade formic acid were purchased from Fisher Scientific, Ltd. (Loughborough, UK). The positive control drugs for genotoxicity tests were obtained from Sigma-Aldrich Co. (St. Louis, MO, USA). An Aroclor1254 (0.5 g/kg) pretreated rat liver S9 (Molecular Toxicology, Inc, Boone, NC, USA) and a cofactor-I (Oriental Yeast Co., Ltd., Itabashi, Tokyo, Japan) were prepared for the S9 mix.

### 2.2. Preparation of Noni Fruit and Seed Substances/Samples

Noni at the mature stage was cultivated on Java Island, Indonesia, processed into frozen and dried whole fruits and dried seeds, and imported through Nongbuae (Icheon, Korea) and Cosmo International (Icheon, Korea). Their morphological identification was carefully validated by Dr. JH Paik, Korea Research Institute of Bioscience and Biotechnology (KRIBB) and Dr. GY Choi, Korea Institute of Oriental Medicine (KIOM), Korea. A voucher specimen (KIOM010086) was deposited in the KM Science Research Division of KIOM.

For the preparation of the puree of noni fruit (PNF), the frozen fruit was washed briefly and processed into a puree after removal seeds and skin via a micro-mesh screen, then pasteurized at 80 ± 5 °C for 1 h at fruit processing facility in Icheon, Republic of Korea. The puree was concentrated to dryness in a spray-drying system (PNF, 52.8 kg, 36.3% yield).

For the preparation of the aqueous extract of noni fruit (EDNF), the dried whole noni was chopped and extracted with distilled water in a crude extraction system (95 ± 5 °C, 3 h, 2 times) according to methods previously described in the traditional Chinese and Korea medicine [29]. Insoluble particles were removed using a 100-mesh strainer. The filtrates were concentrated in an evaporator system at 40 °C and dried with a spray-dryer, then pasteurized at 90 ± 5 °C for 0.5 h. The final dried extracts were EDNF (51.8 kg, 9.7% yield).

To prepare the powder of dried noni fruit (PDNF) and the powder of dried noni seed (PDNS), dried whole fruit and seeds were ground, passed through a 120-mesh sieve, and then repeatedly passed through a metal dust remover equipped with 10,000 Gauss magnetic rods (68 pieces) to remove the iron powder that could be mixed in the grinding process. The ground powders PDNF (15.7 kg, 78.4% yield) and PDNS (18.0 kg, 81.8% yield) were sterilized using the retort method at 121 °C for 0.5 h and stored in desiccated condition.

### 2.3. Chromatographic Analysis

By analyzing the constituents of each sample, the quality control was assessed with an HPLC-diode array detector (DAD) system (1260 infinity, Agilent Technologies, Santa Clara, CA, USA) including a Luna Omega C18 column (2.1 × 100 mm, 1.6 μm, Phenomenex, Torrance, CA, USA). The noni samples and the reference chemicals (DAA, AA, and 5-HMF) were prepared at 10 mg/mL and 0.05 mg/mL in 50% (*v/v*) methanol in distilled water. The samples were analyzed using a sequential gradient mobile phase system from a 99:1 mixture to a 50:50 mixture of water containing 0.1% (*v/v*) formic acid (mobile phase A) and acetonitrile (mobile phase B) for 30 min at a flow rate of 0.2 mL/min. The detected signals on the chromatograms were monitored using a DAD detector with a spectral range of 230–320 nm.

### 2.4. Animals

The animal experimental protocol for micronucleus testing was approved by the Institutional Animal Care and Use Committee of the Korea Testing & Research Institute (Hwasun, Korea). Specific pathogen-free CrlOri:CD1(ICR) mice (7 weeks old) were supplied by OrientBio (Gapyeong, Korea). They were housed in a polysulfonate cage (<5 mouse/cage) under the strically controlled conditions: temperature, 21 ± 1 °C; relative humidity, 53.3 ± 10%; 12 h day/12 h night cycle. All the animals had free access to standard rodent chow and tap water.

### 2.5. Genotixicity Tests

The three battery of standard genotoxicity tests were conducted according to the OECD guidelines for the Testing of chemicals, TG 471 Bacterial Reverse Mutation Test [30], TG 473 in vitro Mammalian Chromosome Aberration Test [31], TG 474 Mammalian Erythrocyte Micronucleus Test [32], and the Ministry of Food and Drug Safety (MFDS) Standard Guidelines for Toxicity Study of Pharmaceuticals (MFDS Notification No. 2017-71) [33].

#### 2.5.1. Ames Test

The experimental procedure was modified as previously described [34]. Briefly, to determine the solubility and toxicity of each substance during bacterial growth, a preliminary dose range-finding test was conducted, resulting in a dose of 5000 μg/plate being chosen as the maximum test dose. Four Salmonella typhimurium strains (His auxotrophic mutant) and one Escherichia coli strain (Trp auxotrophic mutant) were used for the test. Bacterial strains were exposed to each substance (0–5000 μg/plate) in the mixes with or without of the metabolic activator S9 (final 5%, *v*/*v*). 2-Aminoanthracene (2-AA, 2.0 μg/plate) for TA1535, TA1537, and WP2 uvrA and Benzo[a]pyrene (B[a]P, 2.5 μg/plate) for TA98 and TA100 in the presence of metabolic activation were used as positive controls, respectively. Furylfuramide (AF-2, 0.01 μg/plate) was used as a positive control for TA100, TA98, and WP2 uvrA. Sodium azide (SA, 0.5 μg/plate) for TA1535 and 9-aminoacridine (9-AA, 40.0 μg/plate) for TA1537 were used as positive controls, in the absence of metabolic activation. Sterile distilled water was used as a negative control irrespective of the presence of metabolic activation.

#### 2.5.2. Chromosomal Aberration Test

The chromosomal aberration test was conducted according to the slightly modified methods as previously described [35] using cultured mammalian Chinese hamster lung (CHL)/IU cells. In order to determine the solubility and toxicity of each substance, a preliminary dose range-finding test was conducted. The relative increase in cell count (RICC) was determined using the following formula:

$$\mathrm{RICC}\;\left(\%\right)=\frac{\mathrm{increase}\;\mathrm{in}\;\mathrm{number}\;\mathrm{of}\;\mathrm{cells}\;\mathrm{in}\;\mathrm{treated}\;\mathrm{cultures}\;\left(\mathrm{final}-\mathrm{starting}\right)}{\mathrm{increase}\;\mathrm{in}\;\mathrm{number}\;\mathrm{of}\;\mathrm{cells}\;\mathrm{in}\;\mathrm{negative}\;\mathrm{control}\;\mathrm{cultures}\;\left(\mathrm{final}-\mathrm{starting}\right)}\times 100$$

The maximum dose of each noni substance was chosen at which the RICC values were ≥55%. CHL/IU cells were subjected to each substance, mitomycin C (MMC, 0.1 mg/mL) or cyclophosphamide (CPA, 5 mg/mL), for a 6 h treatment and 18 h of recovery in the presence (6–18[+]) or absence (6–18[−]) of the metabolic activator S9 mix (final 3%, *v*/*v*). In parallel, the cells were also subjected to each substance or MMC (0.05 mg/mL) for a 24 h treatment in the absence of the metabolic activator S9 mix (24–0[−]). After 24 h, for accumulation at metaphase, the cells were treated with colcemide (0.2 μg/mL) for 2 h for the accumulation of cells at the metaphase. The cells were collected, treated with 75 mM KCl, and fixed in a mixture of cold ethanol: acetic acid (3:1, *v*/*v*). Dried smears were stained with 5% (*v*/*v*) Giemsa solution. One hundred and fifty metaphases per smear were counted using the blind method under a microscope (1000×). Chromosomal aberrations were categorized as follows; chromosome-type break (ctb), chromosome-type exchange (cte), chromatid-type break (csb), chromatid-type exchange (cse), and others showing metaphase with more than 10 aberrations including gaps (multiple aberrations) or with chromosome fragmentation. Statistical analyses were performed using SPSS Statistics 19 (IBM, New York, NY, USA) for Medical Sciences. To compare the drug treatment groups with the vehicle-treated group, Fisher’s exact test was carried out. The dose–response analysis was performed by linear-by-linear association of the χ^2^ test.

#### 2.5.3. Micronucleus Test

To detect damage to the chromosomes or the mitotic apparatus of erythroblasts, an in vivo micronucleus test was conducted using a slightly modified method of Erexson [36]. In the preliminary test, the oral administration of each substance at 0–2000 mg/kg/d for 2 d did not cause any signs of toxicity in ICR mice. Based on this result, five ICR male mice per group received 500–2000 mg/kg/d of each substance via oral gavage twice. CPA (70 mg/kg) was intraperitoneally administered to mice 24 h before sacrifice, as a positive control. Twenty-four hours after the final drug treatment, bone marrow cells were prepared by flushing the femurs and smeared on a glass slide. The slides were dried and stained with acridine orange (40 μg/mL). Then, micronuclei were counted under a fluorescent microscope (Eclipse, Nikon, Tokyo, Japan) at 400× magnification and their morphological identification followed Hayashi’s method [37]. Micronuclei appeared as green dots with a red background, polychromatic erythrocytes (PCEs) appeared red, and normochromatic erythrocytes (NCEs) showed as dark gray. The genotoxic index in one animal was indicated as the number of micronucleated polychromatic erythrocytes (MNPCEs) in 4000 PCEs. The results were assessed using Lovell’s method [38]. Statistical analyses were conducted using SPSS Statistics 19. Analysis of one-way variance and Dunnett’s tests were used to compare the PCE:RBC ratios and body weights among the treatment groups. Student’s t-test was used to analyze the mean differences between the negative and positive controls. The non-parametric Kruskal–Wallis H test was used to compare the frequency of micronuclei among the treatment groups. The Mann–Whitney U test was used to analyze negative and positive control data.

## 3. Results

### 3.1. Phytochemical Analysis

**Figure 1:**
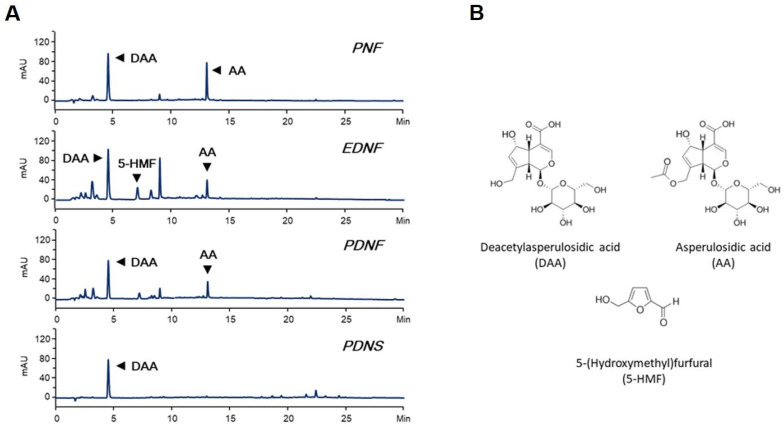
(**A**) Chromatograms of noni fruit and seed substances; (**B**) the structures of the major constituents. The reveled signals on chromatograms were detected at 245 nm.

As shown in the HPLC-DAD chromatogram of each sample, the major constituents were DAA and AA in the PNF and PDNF; DAA, AA, and 5-HMF in the EDNF; and DAA in the PDNS (Figure 1). The retention times (Rt) and contents of the main constituents in each sample are shown in Table 1 and indicate consistency in the quality of each sample. These results are consistent with a previous report that the two iridoids, DAA and AA, can be found in noni fruit and leaf samples from different geographical regions and can thus serve as useful identity markers for authentic noni fruit and leaf products [39,40].

### 3.2. Ames Test

**Figure 2:**
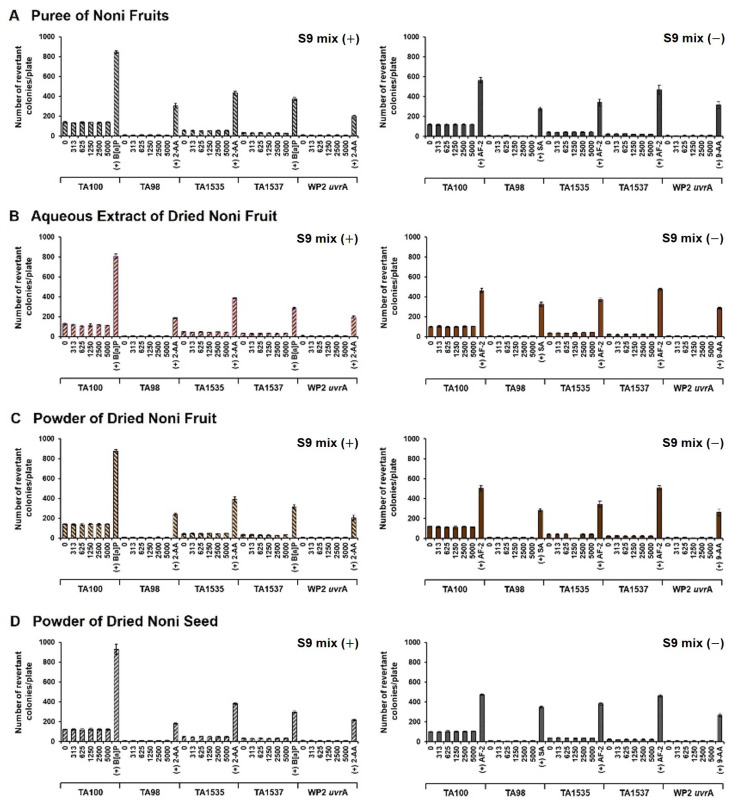
Summary of the Ames test of noni fruit preparations in Salmonella typhimurium (TA98, TA100, TA1535, and TA1537) and Escherichia coli (WP2 uvrA) in the presence or absence of S9 mix. All doses of each substance and the control were analyzed in triplicate.

We confirmed that there was no growth inhibition or precipitation and no bacterial colonies in the experimental solutions prepared with the S9 mix. All positive control drugs remarkably increased the number of revertant bacterial colonies under our tested conditions, which confirmed the validity of the Ames test system. The mean numbers of revertant bacterial colonies counted in the noni fruit and seed substance-treated groups did not increase compared to the negative control up to 5000 μg/plate, regardless of the presence of metabolic activation (Figure 2).

### 3.3. Chromosomal Aberration Test

To determine the mutagenic potential of noni fruit substances by inducing structural chromosomal aberrations, mammalian CHL/IU cells were exposed to each substance with or without the S9 mix for 6 or 24 h. There was no turbidity or precipitation of the substance preparations at any of the test doses. The frequencies of metaphasic chromosomes in the cultured CHL cells showing structural or numerical aberrations were less than 2% in the negative control, irrespective of the presence of the S9 mix. The positive control drugs CPA (+S9 mix) and MMC (−S9 mix) increased the frequencies of CHL/IU cells showing chromosomal structural abnormalities by more than 40%, indicating the validity of our test system (Table 2, Table 3, Table 4 and Table 5). Among 150 metaphase cells per sample, the proportions of numerical aberration were less than 5% at all doses of noni substances: up to 5000 μg/mL PNF, 450 μg/mL PDNS, 4650 μg/mL EDNF, and 4100 µg/mL PDNF after 6 h treatment in the presence (6–18[+]) or absence (6–18[−]) of the S9 mix, and after 24 h treatment and 0 h of recovery in the absence of the S9 mix (24–0[−]) (Table 5). At all tested doses of each noni fruit or seed substance, the frequencies of the cells showing that the structural aberrations were less than 3%, and that the number of total cells with structural aberrations excluding gaps (gap^−^) was below 5%. Therefore, according to the criteria of Sofuni [41], these results demonstrate that noni fruits and seed substances do not exert mutagenic potential to induce chromosomal damage under the present experimental conditions.

### 3.4. In Vivo Micronucleus Test

Male ICR mice receiving noni substances at 500–2000 mg/kg/d was monitored for five days. The experimental mice did not show any toxicity-related signs, such as loss of body weight (data not shown) or mortality (Table 6, Table 7, Table 8 and Table 9). A genotoxic index expressed as the mean percentage of MNPCEs in PCEs by counting a total of 4000 PCEs per mouse and a cytotoxic index expressed as the mean percentage were calculated from the average ratio of PCE to RBCs by counting a total of 500 RBCs. As shown in Table 6, Table 7, Table 8 and Table 9, the frequencies of MNPCEs at 2000 mg/kg/d of noni substances (0.02 ± 0.03 to 0.05 ± 0.04) were not increased compared to the negative control (0.04 ± 0.05 to 0.07 ± 0.03) which are within the range of historical background data (data not shown). However, CPA, a positive control, significantly increased the frequency of MNPCEs (6.45 ± 0.31 to 6.81 ± 0.26) as expected. These results demonstrate that none of the noni fruit substances induced micronuclei in the bone marrow cells of male ICR mice under the test conditions.

## 4. Discussion

The recent growing popularity of noni fruits has drawn the attention of the food industry to the development of various noni fruit products, including fruit puree and juice, leached tea, and powder, and to their use in a wide variety of applications including a herbal medicine and a chemical reagent. Accordingly, much attention has been paid to the toxicity of noni fruits. Among the various phytochemicals of noni fruits, anthraquinones, such as lucidin, rubiadin, and alizarin, are known to have genotoxic and carcinogenic potential [24,25,42]. It has been reported that noni fruit seed-free puree and pureed products (juices and capsules) do not contain detectable amounts of anthraquinones, but products containing fruit peel and seeds were found to contain significant amounts of anthraquinones [15]. Therefore, the possible contents of anthraquinones in noni preparations are thought to induce genotoxicity. In this study, we evaluated the genotoxicity of noni preparations, including the PNF, EDNF, PDNF, and PDNS, using a battery of tests, including in vitro Ames, in vitro chromosomal aberration, and in vivo bone marrow micronucleus tests.

The Ames test is the most frequently employed initial screening method to determine the mutagenic potential of herbal medicines, particularly point mutations [30,43]. In the present study, none of the noni fruit and seed substances, including PDNF, EDNF, PDNF, and PDNS, showed any frameshift mutations or base-pair substitution mutagenic potential, regardless of metabolic activation up to 5000 μg/plate. The results demonstrated that noni fruits and seeds have no mutagenic potential toward any genes related to point mutations under the all the tested conditions.

According to the updated guidelines to improve the reliability of the test, acceptable cytotoxicity levels were identified using a preliminary range-finding test. In this test, the frequencies of both structural and numerical aberrations of four noni substances, PDNF, EDNF, PDNF, and PDNS, were less than 2% at all test concentrations, irrespective of metabolic activation, and the data did not show a statistically significant increase or dose dependency. None of the results were included in the historical negative control data. Increased abnormal changes in the structure or number of chromosomes are related to tumor progression [43]. Despite reports that noni seeds and skin contain anthraquinones, which may cause genotoxicity [15,26], in this study, there was no change in the structure or number of chromosomal abnormalities, not only in the whole fruit aqueous extracts and powders, but also in the seed powders. Additionally, noni substances contain high contents of DAA (iridoids) that are antigenotoxic reagent [44]. Therefore, non-genetoxicity of noni substances may relate to the presence of iridoid and the absence of anthraquinones. Taken together, our results show that noni seed and fruit preparations do not appear to mutate any genes in cultured CHL cells under the experimental conditions.

The in vivo micronucleus test is the most commonly used in vivo assay (OECD TG474) [32] and is useful for detecting chemically induced chromosomal damage and accumulated genotoxic damage in response to environmental risk [45,46]. Increased micronucleus frequency is correlated with carcinogenicity [47,48]. The micronucleus assay is highly reliable and rapid, and it can determine a broad spectrum of DNA damage at the chromosomal level, particularly for the assessment of mutagenic risk [49,50]. In the present study, the mice administered with noni substances do not show mortality up to 2000 mg/kg body weight (b.w). These results are consistent with reports that the acute lethal doses (LD_50_) of orally injected noni fruit and seed substances are greater than 15,000 mg/kg and 5000 mg/kg b.w., respectively [44,51]. There were no significant or dose-related changes in the genotoxic index, MNPCE/PCEs, or the cytotoxic index, PCE/RBC (PCE + NCE) ratio, at any tested doses (0–2000 mg/kg/d) of noni substances, compared to the negative control. The results demonstrate that the four noni substances do not have the potential to induce clastogenicity and aneuploidy in bone marrow cells up to 2000 mg/kg/d under the conditions tested. This suggests that noni fruits and seeds are unlikely to cause genetic damage or carcinogenicity. However, different genotoxic effects have been observed between distinct preparations from the same plants. For example, the chloroform extract of *Aloe vera* showed genotoxicity in mitotic index and chromosome aberration tests, but their crude extract and hydroalcoholic extract showed negative results at the same or even higher doses [52,53,54]. Therefore, a detailed study is required to evaluate the genotoxicity of noni fruits and seeds under diverse experimental conditions.

## 5. Conclusions

Our results of genotoxicity studies, including an in vitro Ames test, a chromosomal aberration test, and an in vivo micronucleus test, indicate that noni fruits and seeds are not mutagenic or clastogenic. This suggests that noni can be safely used as a potential therapeutic agent for nutraceutical and pharmaceutical development, at least within the dose range demonstrated in the present study.

## Figures and Tables

**Table 1:** Contents of the major constituents in noni fruit and seed substances.

		PNF	EDNF	PDNF	PDNS
**DAA**	Rt (min)	4.59 ± 0.05	4.56 ± 0.01	4.57 ± 0.02	4.53 ± 0.02
	Contents (mg/g)	5.70 ± 0.01	9.44 ± 0.02	4.67 ± 0.01	4.13 ± 0.01
**AA**	Rt (min)	13.06 ± 0.01	12.98 ± 0.02	13.07 ± 0.01	_
	Contents (mg/g)	5.20 ± 0.01	3.52 ± 0.01	2.19 ± 0.01	_
**5-HMF**	Rt (min)	_	7.08 ± 0.04	_	_
	Contents (mg/g)	_	3.23 ± 0.01	_	_

Rt, retention time; PNF, puree of noni fruits; EDNF, aqueous extract of dried noni fruits; PDNF, powder of dried noni fruits; and PDNS, powder of dried noni seeds; DAA, deacetylasperulosidic acid; AA, asperulosidic acid; 5-HMF, 5-hydroxymethylfulrfural.

**Table 2:** Results of the chromosomal aberration test of the puree of noni fruits (PNF).

Drug	Dose (μg/mL)	Chromosomal Aberration								Numerical ab.	RICC (%)
		Structural ab.									
		Chromatid Type		Chromosome Type		Gaps	Others	Total			
		ctb	cte	csb	cse	Gaps	Others	−Gaps	+Gaps	PP + ER	
*Treatment: 6 h treatment and 18 h recovery (−S9 mix)*											
PNF	0	0	0	0	0	0	0	0	0	0	100
	1250	0	0	0	0	1	0	0	1	0	88
	2500	0	0	0	0	0	0	0	0	0	89
	5000	0	0	0	0	0	0	0	0	0	79
MMC	0.1	9	32	4	0	1	0	45 23 **/22	46	0	72
*Treatment: 6 h treatment and 18 h recovery (+S9 mix)*											
PNF	0	0	1	0	0	0	0	1	1	0	100
	1250	0	0	0	0	0	0	0	0	0	96
	2500	0	1	0	0	0	0	1	1	0	96
	5000	0	0	0	0	1	0	0	0	0	93
CPA	5.0	5	42	1	0	1	0	48 25 */23	49	0	60
*Treatment: 24 h treatment and 0 h recovery (−S9 mix)*											
PNF	0	0	0	0	0	0	0	0	0	0	100
	1250	0	0	2	0	0	0	2	2	0	86
	2500	0	0	0	0	0	0	0	0	0	84
	5000	0	0	0	0	0	0	0	0	0	78
MMC	0.05	4	36	2	1	0	0	42 23 */19	42	0	63

ctb, chromatid-type break; cte, chromatid-type exchange; csb, chromosome type break; cse, chromosome type exchange; PP + ER, polyploid + endoreduplication; others, metaphases with more than 10 aberrations (including gaps) or with chromosome fragmentation; and gaps, chromatid type + chromosome type gaps. Micomycin C (MMC) and cyclophosphamide (CPA), positive controls. The data are presented as the mean number of chromosomal aberrations in 150 metaphases from duplicate experiments. * *p* < 0.05 and ** *p* < 0.01.

**Table 3:** Results of the chromosomal aberration test of aqueous extract of dried noni fruits (EDNF).

Drug	Dose (μg/mL)	Chromosomal Aberration								Numerical ab.	RICC (%)
		Structural ab.									
		Chromatid Type		Chromosome Type		Gaps	Others	Total			
		ctb	cte	csb	cse	Gaps	Others	−Gaps	+Gaps	PP + ER	
*Treatment: 6 h treatment and 18 h recovery (−S9 mix)*											
EDNF	0	0	0	0	0	1	0	0	1	0	100
	1162.5	0	0	0	0	0	0	0	0	0	93
	2325	0	0	0	0	0	0	0	0	0	88
	4650	0	1	0	0	0	0	0	0	0	57
MMC	0.1	8	45	4	1	1	0	55 29 **/26	56	0	65
*Treatment: 6 h treatment and 18 h recovery (+S9 mix)*											
EDNF	0	0	0	0	0	0	0	0	0	0	100
	1250	0	0	0	0	0	0	0	0	0	90
	2500	0	0	0	0	1	0	0	1	0	84
	5000	0	0	0	0	0	0	0	0	0	66
CPA	5.0	9	43	3	1	1	1	57 30 */27	58	0	55
*Treatment: 24 h treatment and 0 h recovery (−S9 mix)*											
EDNF	0	2	0	0	0	0	0	2	2	0	100
	1085.5	0	1	0	0	0	0	0	0	0	95
	2175	0	0	0	0	0	0	0	0	0	87
	4350	0	0	0	0	0	0	0	0	0	57
MMC	0.05	8	43	5	2	0	0	58 29 */29	58	0	60

ctb, chromatid-type break; cte, chromatid-type exchange; csb, chromosome type break; cse, chromosome type exchange; PP + ER, polyploid + endoreduplication; others, metaphases with more than 10 aberrations (including gaps) or with chromosome fragmentation; and gaps, chromatid type + chromosome type gaps. Micomycin C (MMC) and cyclophosphamide (CPA), positive controls. The data are presented as the mean number of chromosomal aberrations in 150 metaphases from duplicate experiments. * *p* < 0.05 and ** *p* < 0.01.

**Table 4:** Results of the chromosomal aberration test of powder of dried noni fruits (PDNF).

Drug	Dose (μg/mL)	Chromosomal Aberration								Numerical ab.	RICC (%)
		Structural ab.									
		Chromatid Type		Chromosome Type		Gaps	Others	Total			
		ctb	cte	csb	cse	Gaps	Others	−Gaps	+Gaps	PP + ER	
*Treatment: 6 h treatment and 18 h recovery (−S9 mix)*											
PDNF	0	0	1	0	0	1	0	1	2	0	100
	512.5	0	0	0	0	0	0	0	0	0	86
	1025	0	0	0	0	0	0	0	0	0	79
	2050	0	0	0	0	0	0	0	0	0	78
	4100	0	0	0	0	0	0	0	0	0	57
MMC	0.1	7	35	3	2	0	0	47 24 **/23	47	0	66
*Treatment: 6 h treatment and 18 h recovery (+S9 mix)*											
PDNF	0	0	0	0	0	0	0	0	0	0	100
	625	0	1	0	0	0	0	1	1	0	95
	1250	0	1	0	0	0	0	1	1	0	91
	2500	0	0	0	0	0	0	0	0	0	58
CPA	5.0	5	39	2	0	0	0	46 23 */23	46	0	64
*Treatment: 24 h treatment and 0 h recovery (−S9 mix)*											
PDNF	0	0	1	0	0	0	0	1	2	0	100
	275	0	0	0	0	2	0	0	2	0	84
	550	0	0	0	0	0	0	0	0	0	79
	1100	0	0	0	0	0	0	0	0	0	57
MMC	0.05	6	40	2	0	0	0	48 24 */24	48	0	76

ctb, chromatid-type break; cte, chromatid-type exchange; csb, chromosome type break; cse, chromosome type exchange; PP + ER, polyploid + endoreduplication; others, metaphases with more than 10 aberrations (including gaps) or with chromosome fragmentation; and gaps, chromatid type + chromosome type gaps. Micomycin C (MMC) and cyclophosphamide (CPA), positive controls. The data are presented as the mean number of chromosomal aberrations in 150 metaphases from duplicate experiments. * *p* < 0.05 and ** *p* < 0.01.

**Table 5:** Results of the chromosomal aberration test of powder of dried noni seeds.

Drug	Dose (μg/mL)	Chromosomal Aberration								Numerical ab.	RICC (%)
		Structural ab.									
		Chromatid Type		Chromosome Type		Gaps	Others	Total			
		ctb	cte	csb	cse	Gaps	Others	−Gaps	+Gaps	PP + ER	
*Treatment: 6 h treatment and 18 h recovery (−S9 mix)*											
PDNS	0	0	1	0	0	0	0	1	1	0	100
	112.5	0	0	0	0	1	0	0	1	0	94
	225	0	0	0	0	0	0	0	0	0	70
	450	0	1	0	0	0	0	0	0	0	51
MMC	0.1	6	41	6	2	1	0	55 27 **/28	56	0	71
*Treatment: 6 h treatment and 18 h recovery (+S9 mix)*											
PDNS	0	0	1	0	0	0	0	1	1	0	100
	525	0	0	0	0	0	0	0	0	0	82
	1050	0	0	0	0	0	0	0	0	0	69
	2100	0	1	0	0	0	0	1	1	0	57
CPA	5.0	7	45	6	1	0	1	60 32 */28	60	0	63
*Treatment: 24 h treatment and 0 h recovery (−S9 mix)*											
PDNS	0	0	0	0	0	1	0	0	1	0	100
	92.5	0	0	0	0	0	0	0	0	0	83
	185	0	0	0	0	0	0	0	0	0	66
	370	0	2	0	0	0	0	2	2	0	55
MMC	0.05	9	41	3	0	0	0	53 25 */28	53	0	83

Ctb, chromatid-type break; cte, chromatid-type exchange; csb, chromosome type break; cse, chromosome type exchange; PP + ER, polyploid + endoreduplication; others, metaphases with more than 10 aberrations (including gaps) or with chromosome fragmentation; and gaps, chromatid type + chromosome type gaps. Micomycin C (MMC) and cyclophosphamide (CPA), positive controls. The data are presented as the mean number of chromosomal aberrations in 150 metaphases from duplicate experiments. * *p* < 0.05 and ** *p* < 0.01.

**Table 6:** Results of the micronucleus test in ICR mice exposed to oral treatment of puree of noni fruits (PNF).

Drug	Dose (mg/kg/d)	Mortality (Dead/Total)	MNPCE/4000 PCEs (%, Mean ± S.D.)	PCE/(PCE + NCE) (%, Mean ± S.D.)
PNF	0	0/5	0.07 ± 0.03	50.44 ± 0.91
	500	0/5	0.06 ± 0.04	50.68 ± 0.43
	1000	0/5	0.03 ± 0.03	50.87 ± 0.36
	2000	0/5	0.02 ± 0.03	50.84 ± 0.43
CPA	70	0/5	6.45 ± 0.31 *	46.91 ± 0.72 *

Five male ICR mice per group were administered noni substance or cyclophosphamide (CPA) as a positive control. MNPCE, micronucleated polychromatic erythrocytes; PCE, polychromatic erythrocytes; and NCE, normochromatic erythrocytes. Data are presented as the mean ± S.D. * *p* < 0.05.

**Table 7:** The results of the micronucleus test in ICR mice exposed to oral treatment of aqueous extract of dried noni fruits (EDNF).

Drug	Dose (mg/kg/d)	Mortality (Dead/Total)	MNPCE/4000 PCEs (%, Mean ± S.D.)	PCE/(PCE + NCE) (%, Mean ± S.D.)
EDNF	0	0/5	0.05 ± 0.04	49.97 ± 0.67
	500	0/5	0.05 ± 0.04	50.38 ± 1.07
	1000	0/5	0.04 ± 0.03	50.67 ± 0.59
	2000	0/5	0.04 ± 0.03	51.00 ± 0.56
CPA	70	0/5	6.79 ± 0.27 *	46.14 ± 0.58 *

Five male ICR mice per group were administered noni substance or cyclophosphamide (CPA) as a positive control. MNPCE, micronucleated polychromatic erythrocytes; PCE, polychromatic erythrocytes; and NCE, normochromatic erythrocytes. Data are presented as the mean ± S.D. * *p* < 0.05.

**Table 8:** The results of the micronucleus test in ICR mice exposed to oral treatment of powder of dried noni fruits (PDNF).

Drug	Dose (mg/kg/d)	Mortality (Dead/Total)	MNPCE/4000 PCEs (%, Mean ± S.D.)	PCE/(PCE + NCE) (%, Mean ± S.D.)
PDNF	0	0/5	0.04 ± 0.05	51.37 ± 0.71
	500	0/5	0.07 ± 0.04	51.18 ± 0.61
	1000	0/5	0.06 ± 0.02	51.40 ± 0.28
	2000	0/5	0.03 ± 0.03	50.94 ± 0.36
CPA	70	0/5	6.47 ± 0.28 *	47.07 ± 0.99 *

Five male ICR mice per group were administered noni substance or cyclophosphamide (CPA) as a positive control. MNPCE, micronucleated polychromatic erythrocytes; PCE, polychromatic erythrocytes; and NCE, normochromatic erythrocytes. Data are presented as the mean ± S.D. * *p* < 0.05.

**Table 9:** Results of the micronucleus test in ICR mice exposed to oral treatment of powder of dried noni seeds (PDNS).

Drug	Dose (mg/kg/d)	Mortality (Dead/Total)	MNPCE/4000 PCEs (%, Mean ± S.D.)	PCE/(PCE + NCE) (%, Mean ± S.D.)
PDNS	0	0/5	0.06 ± 0.04	50.62 ± 0.41
	500	0/5	0.05 ± 0.05	50.45 ± 1.27
	1000	0/5	0.06 ± 0.03	51.22 ± 0.45
	2000	0/5	0.05 ± 0.03	50.78 ± 0.65
CPA	70	0/5	6.81 ± 0.26 *	46.18 ± 1.26 *

Five male ICR mice per group were administered noni substance or cyclophosphamide (CPA) as a positive control. MNPCE, micronucleated polychromatic erythrocytes; PCE, polychromatic erythrocytes; and NCE, normochromatic erythrocytes. Data are presented as the mean ± S.D. * *p* < 0.05.

## Data Availability

The data are available from the corresponding author.
